# Biogenic VOCs Emission Profiles Associated with Plant-Pest Interaction for Phenotyping Applications

**DOI:** 10.3390/s22134870

**Published:** 2022-06-28

**Authors:** Milton Valencia-Ortiz, Afef Marzougui, Chongyuan Zhang, Sapinder Bali, Steven Odubiyi, Vidyasagar Sathuvalli, Nilsa A. Bosque-Pérez, Michael O. Pumphrey, Sindhuja Sankaran

**Affiliations:** 1 Department of Biological System Engineering, Washington State University, Pullman, WA 99164, USA; m.valenciaortiz@wsu.edu (M.V.-O.); afef.marzougui@wsu.edu (A.M.); chongyuan.zhang@wsu.edu (C.Z.); 2 Department of Plant Pathology, Washington State University, Pullman, WA 99164, USA; sapinder.bali@wsu.edu; 3 Department of Entomology, Plant Pathology and Nematology, University of Idaho, Moscow, ID 83844, USA; stevenodubiyi@uidaho.edu (S.O.); nbosque@uidaho.edu (N.A.B.-P.); 4 Department of Crop and Soil Science, Hermiston Agricultural Research & Extension Center, Oregon State University, Hermiston, OR 97838, USA; vidyasagar@oregonstate.edu; 5 Department of Crop and Soil Sciences, Washington State University, Pullman, WA 99164, USA; m.pumphrey@wsu.edu

**Keywords:** biotic stress, biomarkers, GC-FID, nematode, Hessian fly, potato, wheat

## Abstract

Pest attacks on plants can substantially change plants’ volatile organic compounds (VOCs) emission profiles. Comparison of VOC emission profiles between non-infected/non-infested and infected/infested plants, as well as resistant and susceptible plant cultivars, may provide cues for a deeper understanding of plant-pest interactions and associated resistance. Furthermore, the identification of biomarkers—specific biogenic VOCs—associated with the resistance can serve as a non-destructive and rapid tool for phenotyping applications. This research aims to compare the VOCs emission profiles under diverse conditions to identify constitutive (also referred to as green VOCs) and induced (resulting from biotic/abiotic stress) VOCs released in potatoes and wheat. In the first study, wild potato *Solanum bulbocastanum* (accession# 22; SB22) was inoculated with *Meloidogyne chitwoodi* race 1 (Mc1), and Mc1 pathotype Roza (SB22 is resistant to Mc1 and susceptible to pathotype Roza), and VOCs emission profiles were collected using gas chromatography-flame ionization detection (GC-FID) at different time points. Similarly, in the second study, the VOCs emission profiles of resistant (‘Hollis’) and susceptible (‘Alturas’) wheat cultivars infested with Hessian fly insects were evaluated using the GC-FID system. In both studies, in addition to variable plant responses (susceptibility to pests), control treatments (non-inoculated or non-infested) were used to compare the VOCs emission profiles resulting from differences in stress conditions. The common VOC peaks (constitutive VOCs) between control and infected/infested samples, and unique VOC peaks (induced VOCs) presented only in infected/infested samples were analyzed. In the potato-nematode study, the highest unique peak was found two days after inoculation (DAI) for SB22 inoculated with Mc1 (resistance response). The most common VOC peaks in SB22 inoculated with both Mc1 and Roza were found at 5 and 10 DAI. In the wheat-insect study, only the Hollis showed unique VOC peaks. Interestingly, both cultivars released the same common VOCs between control and infected samples, with only a difference in VOC average peak intensity at 22.4 min retention time where the average intensity was 4.3 times higher in the infested samples of Hollis than infested samples of Alturas. These studies demonstrate the potential of plant VOCs to serve as a rapid phenotyping tool to assess resistance levels in different crops.

## 1. Introduction

Climate change strongly influences pest population dynamics that may increase crop damage and new strategies are needed to face challenges from pests and pathogens [1]. In recent years, researchers have focused on volatile organic compounds (VOCs) as a tool to protect plants from stress and boost crop production [2]. These VOCs play essential roles in many ecological functions [3], such as plant defenses to biotic and abiotic stresses, providing information about crop status, mutualists, competitors [4], and promoting plant growth [5]. From an integrated pest management perspective, crop disease resistance is an essential component to protect crop yields against pests and pathogens.

In general, plants under attack activate signaling pathways leading to the expression of plant resistance mechanisms [6]. Gene expression responses and leaf metabolic potential can modify the proportion of VOC biogenesis and emission levels [7]. The VOC biogenesis can be either constitutive or induced [8]. The emission of constitutive VOCs occurs regardless of whether the plant is under stress, while the emission of induced VOCs only occurs under conditions of stress [9]. For example, the release of biogenic VOCs in the grapevine by exogenous stimuli suggests a plant defense response against pathogens, resistance induction, and biomarker use [10]. Therefore, biogenic VOCs can serve as biomarkers to inform about the plant defense mechanisms and resistance levels. Given the potential to detect VOCs rapidly and non-invasively, biomarkers can also assist as a robust phenotyping tool. However, the application of biogenic VOCs for phenotyping is still limited due to their inherent reactivity and low concentrations [11]. There is a need for further evaluation of such concepts to assess the applicability of phenotyping methods to study crop resistance to pests and pathogens. The present study was designed to evaluate the variability in VOC emission profiles as phenotypes using two specific studies involving potato and wheat plants. 

In potato production systems, nematodes limit the crop yield and tuber quality worldwide, where annual losses can be estimated to be around 78 billion USD or 10–15 percent of total yield [12]. Columbia Root-knot nematode (*Meloidogyne chitwoodi*) causes significant damage to potato tubers, affecting the market value in the fresh pack and processing industries. *Solanum bulbocastanum*, a wild potato species, was identified as the first source of genetic resistance to *M. chitwoodi* (Mc1) [13]. The histological analysis of nematode resistance from *S. bulbocastanum (SB22) introgressed into cultivated potato* (PA99N82-4, an advanced breeding clone) indicated restriction in nematode feeding site formation. This resistant response is associated with reactive oxygen species (ROS) accumulation and hypersensitive responses; where salicylic acid, polyamines, and suberin are important resistant mediators [14]. A previous study on PA99N82-4 resistance response found that calcium plays an important role in the hypersensitive response mechanism against *M. chitwoodi* [15]. However, a resistance-breaking pathotype of Mc1, Roza was identified in Prosser, WA [16]. This newly emerged pathotype could successfully penetrate and establish feeding sites in SB22 plants and thus complete its life cycle. In the first study, SB22 plants were inoculated with Mc1 and its pathotype Roza to compare the VOCs emission profiles resulting from resistant and susceptible responses.

In the second study, Hessian fly (*Mayetiola destructor*) infestation in wheat (*Triticum aestivum*) was assessed in resistant (‘Hollis’) and susceptible (‘Alturas’) cultivars. The Hessian fly is a destructive insect affecting wheat in the United States and other wheat-producing regions worldwide [17]. The impact is through canopy and grain losses, and deterioration of the grain quality. The larvae feed on the stem and sap, resulting in low canopy vigor. Moreover, the insect population can grow intensively as several life cycles are supported within a year, thereby making control measures challenging [18]. Thus, Hessian fly resistance in wheat cultivars has proved to be an effective management strategy [19,20]. Sadeghi et al. [21] reported a significant concentration of jasmonic acid in resistant wheat (cv. Hollis) compared to susceptible wheat (cv. Alturas) infested with *M. destructor*. Similarly, Subramanyam et al. [18] reported enhanced production of free polyamines, putrescine, spermidine, and spermine in susceptible cultivars upon Hessian fly infestation in comparison to resistant cultivars and the respective controls. Some of these compounds may be associated with the changes in VOC emission profiles, which need to be evaluated further. 

Although the above-mentioned studies highlight the potential of VOCs as biomarkers, VOC emission profile detection has not been studied in the context of phenotyping and the onset of resistance. The research described in this paper aimed to evaluate the variability in VOC emission profiles in potato and wheat plants resulting from different responses to the pests. Gas chromatography-flame ionization detection (GC-FID) analyses were performed to capture the VOC emission profiles from diverse samples. 

## 2. Materials and Methods

### 2.1. Potato-Nematode Study

SB22 plants were grown from 4-week-old tissue culture seedlings. Eighteen plantlets were transferred from tissue culture media to pre-sterilized 1:1 sand-soil mix and left to grow for 3-weeks in a greenhouse (24 °C/18 °C with 16 h photoperiod) with regular watering. A total of six plants were inoculated with 1000 J2s’ of Mc1 (resistant), another six with 1000 J2s’ of Roza (susceptible), and six plants were used as control (inoculated with sterilized distilled water). The VOCs emission profiles were sampled and analyzed with a GC-FID system at 2, 5, 10, and 25 days after inoculation (DAI). The J2 penetration was confirmed using microscopic evaluation. Details on nematode egg extraction, hatching, and inoculation can be found in Bali et al. [14]

### 2.2. Wheat-Insect Study

The initial plant growth and infestation were performed at the University of Idaho, Moscow, Idaho. Two wheat cultivars, Hollis (resistant) and Alturas (susceptible), were used in this experiment, and plants were infested with Hessian fly. Wheat seeds (15 seeds per pot) were planted in sixteen 10 cm pots filled with soil mixture, where eight pots (4 pots/cultivar × 2 cultivars) were used for infestation with Hessian flies and another eight (4 pots/cultivar × 2 cultivars) were used as control. In addition, two pots containing no plants (were used as blank controls during VOCs emission profiles analysis to eliminate the background noise from other materials). Wheat seedlings were grown at 24 °C with a 16 h photoperiod and irrigated with nutrient water regularly in transparent plexiglass cages (53-cm length × 51-cm width × 51-cm height), which were used to prevent the escape of Hessian flies during the infestation. Adult Hessian flies (five females and four males) were introduced in each cage 11 days after planting eight pots of the wheat seedlings, intended for eggs to hatch and infest the plants. Wheat seedlings were sampled to evaluate VOC emission profiles and analyzed with the GC-FID system at about 18 and 26 days after planting. A more detailed procedure of Hessian fly establishment and infestation for resistant screening can be found in Schotzko and Bosque-Pérez [22].

### 2.3. Sampling and Data Collection

The sampling of VOCs in both studies was performed using headspace sampling using a solid-phase micro-extraction (SPME) fiber. The SPME fiber was made of 0.65 µm polydimethylsiloxane/divinylbenzene (Supelco Co., Bellefonte, PA, USA). For the potato plants, 0.5 g of root tissue was added to 325 g/mL sodium chloride solution, prior to the sampling of the headspace. The method details can be seen in Iyer et al. [23], also described in Marzougui et al. [24]. Data were collected at 2, 5, 10, and 25 DAI. Dynamic headspace sampling with 35 mL/min airflow was used for sampling wheat plants. The analysis was also performed on control (blank) pots without plants. Data were collected at about 18 and 26 days after planting (7 and 15 days after infestation). Since some of the replicates did not have a successful infestation, the datasets across time points were combined to perform a meaningful statistical assessment. Both studies incorporated 50 min sampling time with SPME fibers. The GC-FID system, Hewlett Packard 5890 Series II (Agilent Technologies, Wilmington, DE, USA), was used for analysis. Inlet and detector temperatures were set at 200 °C, respectively. The settings were 33 °C start temperature (5-min hold), ramp rate 2 °C/min to 50 °C and followed by ramp rate 5 °C/min to 225 °C (5-min hold). The GC-FID analysis protocol was similar to those described in Marzougui et al. [24] and Sangjan et al. [25].

### 2.4. Data Analysis

GC-FID data were preprocessed in MATLAB (2021a, The MathWorks, Natick, MA, USA) using a preprocessing protocol developed by Marzougui et al. [24], which includes signal extraction, peak alignment, data matrix reduction, background signals removal (with the help from blank samples), and identification of retention time of peaks that were present in at least two replicates of each condition/treatment. The VOC peaks that occurred only once were not considered for further analysis. R software (release 4.1.1, http://www.r-project.org/ (accessed on 10 January 2022) was then used to present the data in a Venn diagram to visualize the number of common and unique peaks between different treatments within a crop. Common peaks at specific retention time (RTs) refer to the VOC peaks present in both treatments (control and infested), while unique peaks with specific RTs refer to the VOC peaks present only in infected/infested samples. Common and unique peak RTs were extracted using ‘unique’ and ‘setdiff’ functions and presented using the ggplot2 package in the R program. Unpaired *t*-tests were used to compare mean differences in peak intensities of common RTs among treatments. One-way ANOVA and post-hoc Tukey’s test were also used to evaluate the peak intensity comparisons across DAIs. Finally, Python (Version 3.8.0, interpreter—Spyder) was used to arrange the peak intensity and RTs by treatment to display the heatmap of averaging peak intensities across RTs and treatment.

## 3. Results and Discussion

### 3.1. VOC Profiles from Potato Plants

**Figure 1:**
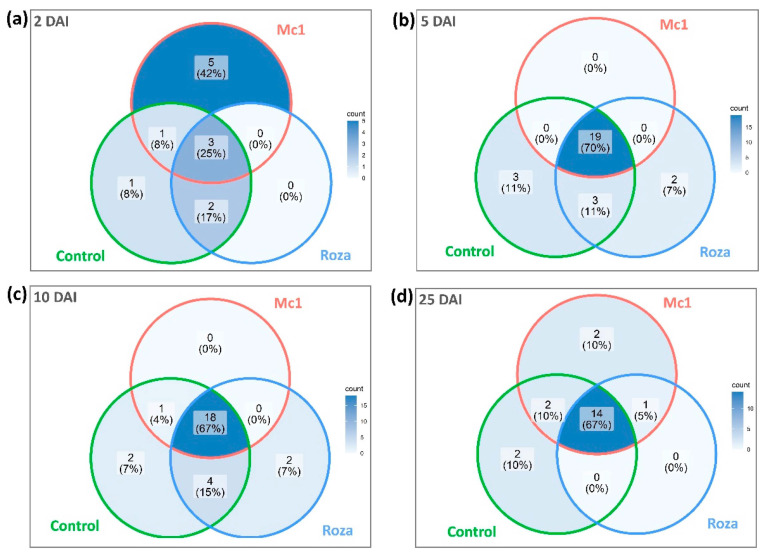
Venn diagrams showing common and unique peaks represented by different retention times extracted from GC-FID data of wild potato species, SB22 plants infected with *M. chitwoodi* Race 1 (Mc1), and its pathotype Roza across different days after inoculation (DAI). (**a**) 2 DAI, (**b**) 5 DAI, (**c**) 10 DAI, and (**d**) 25 DAI.

Plants produce constitutive VOC emissions regardless of stress conditions, while induced VOC emissions occur only under stress conditions [9]. The Venn diagram (Figure 1) developed using GC-FID data provided quantitative data (number of peaks) on constitutive (common peak RTs) and induced volatiles (unique peak RTs). The total number of common and unique peaks RTs representing VOC emission profiles associated with Roza inoculated, Mc1 inoculated, and control samples across different DAI after data preprocessing was 38. The largest number of unique peaks were found at 2 DAI for SB22 inoculated with Mc1 (resistance response) (Figure 1a). This early time point (2 DAI) is critical for SB22 resistance response to Mc1, as, at this time point, the nematode penetrates the root system to try and establish a feeding site. The VOCs emission profiles may indicate the activation of immune responses of the SB22 plants, which subsequently restricts the feeding site formation. Bali et al. [14] reported large differential gene expression in PA99N82-4 (introgression line with nematode resistance from SB22) at 48 h after Mc1 inoculation (also at 7, 14, and 21 DAI), and the VOCs could be associated with the change in the gene expression that majorly represents the defense responses. The histological analysis in the study reported that PA99N82-4 plants were able to restrict the establishment of the feeding site 48 h after inoculation.

**Figure 2:**
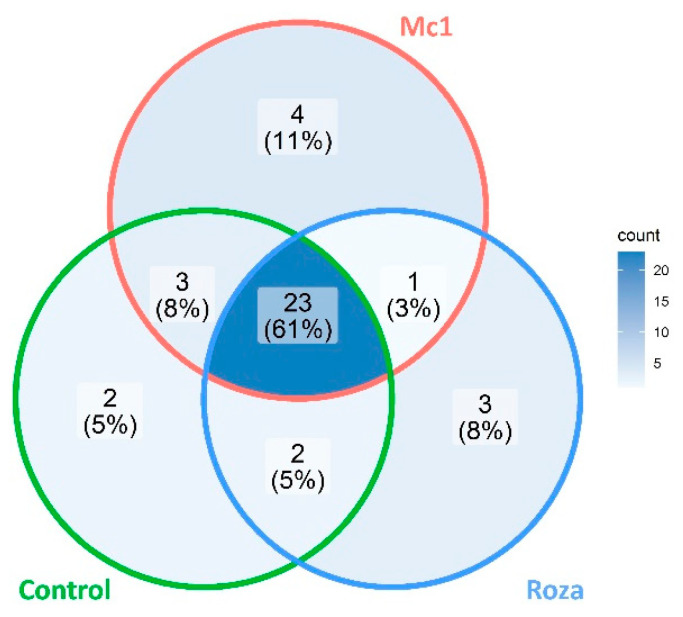
Venn diagram showing common and unique peaks represented by different retention times extracted from GC-FID data of SB22 plants infected with *M. chitwoodi* Race 1 (Mc1) and its pathotype Roza by combining all time points (2, 5, 10, and 25 DAI).

**Figure 3:**
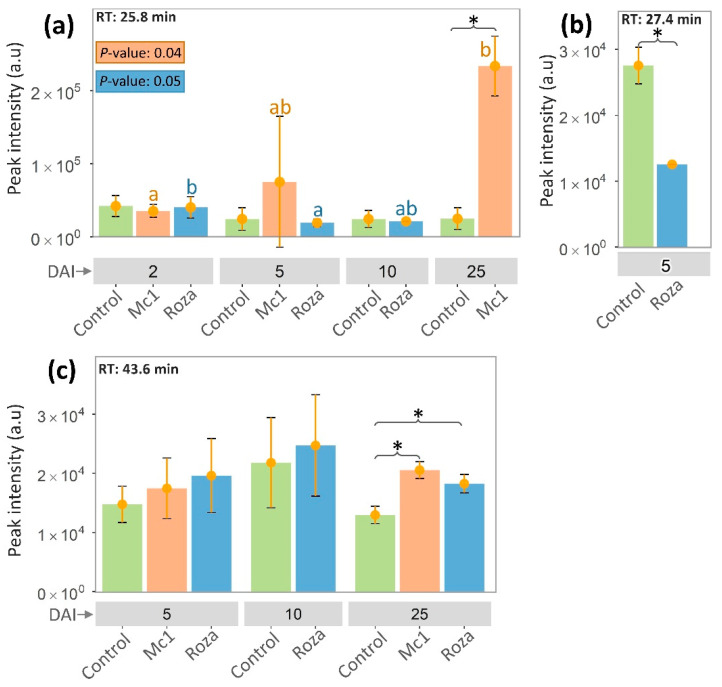
Peak intensity of three common retention times across different days after inoculation of SB22 plants with *M. chitwoodi* Race 1 (Mc1) and its pathotype Roza. Retention time: (**a**) 25.8 min, (**b**) 43.6 min, and (**c**) 27.4 min. * *p*-value < 0.05 from *t*-test analysis. Different letters in each column by nematode race denote significant differences in peak intensities across DAIs at *p* < 0.05 using Tukey’s test.

Most common peaks were found at 5 and 10 DAI (Figure 1b,c) for samples inoculated with both Mc1 and Roza. After combining the VOC peaks data across multiple time points, 23 common peak RTs were detected between Mc1 inoculated, Roza inoculated, and control plants (Figure 2). Out of 23 common peaks RTs, two peaks with RT of 25.8 min and 43.6 min showed significant differences in intensity between Mc1 inoculated and control plants at 25 DAI (Figure 3a,c, *p*-value < 0.05). In addition, the ANOVA of Mc1 VOC peak intensity (25.8 min) indicated significant differences based on the DAIs, where the average peak intensity increased significantly across DAIs (Figure 3a). ANOVA also showed some significant difference in VOC peak intensity (25.8 min) on Roza; however, this average peak intensity did not differ significantly from the control. The average peak intensity of Mc1 plants was 9.5 and 1.6 times those of control at 25.8 min and 43.6 min RTs, respectively. These findings suggest a further increase in constitutive VOC peak intensities as a sign of SB22 defense response to the Mc1 attack. In the case of Roza inoculated plants, out of 23 common peaks RTs, only one peak (43.6 min RT) showed significant differences at 25 DAI (Figure 3c, *p*-value < 0.05) with average peak intensity 1.4 times that of control plants. In contrast, there was a significant reduction (2.2 times, *p*-value < 0.05) in average peak intensity (27.4 min RT) of Roza inoculated plants than those from the control plants at 5 DAI (Figure 3b). The reduction of this VOC peak may potentially be related to the susceptibility of SB22 plants to the Roza and may be associated with the post-infection immune response to nematode attack.

**Figure 4:**
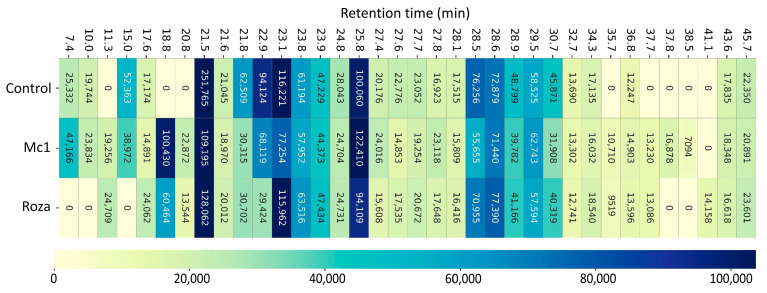
Heat-map showing the peak intensity extracted from GC-FID data of samples from potato SB22 plants infected with *M. chitwoodi* Race 1 (Mc1) and its pathotype Roza by combining all time points (2, 5, 10, and 25 DAI).

Heatmap (Figure 4) of average peak intensities of VOCs across the combined dataset shows variations in VOCs emission profiles between control, Mc1 inoculated, and Roza inoculated SB22 plants. In general, VOC peaks at 21.5, 23.1, and 25.9 RTs showed the highest peak intensities across the samples, although the differences were not statistically significant. 

The literature suggests that VOCs can be indicators of pest/pathogen defense mechanisms. In soybean, Lin et al. [26] reported the sesquiterpene (E,E)-α-farnesene as a major VOC released during nematode infestation. Similarly, increase in α-farnesene and α-bergamotene sesquiterpenes was reported by Castorina et al. [27] in *Vitis vinifera* during nematode *Xiphinema index* (Dagger nematode) attack. In *Arabidopsis thaliana*, the number of nematodes that penetrated roots was reduced by the sesquiterpene nootkatone [28]. Other compounds such as ascaridole and citronellal can also act as toxic compounds against *Meloidogyne incognita* [29].

### 3.2. VOC Profiles from Wheat Plants

**Figure 5:**
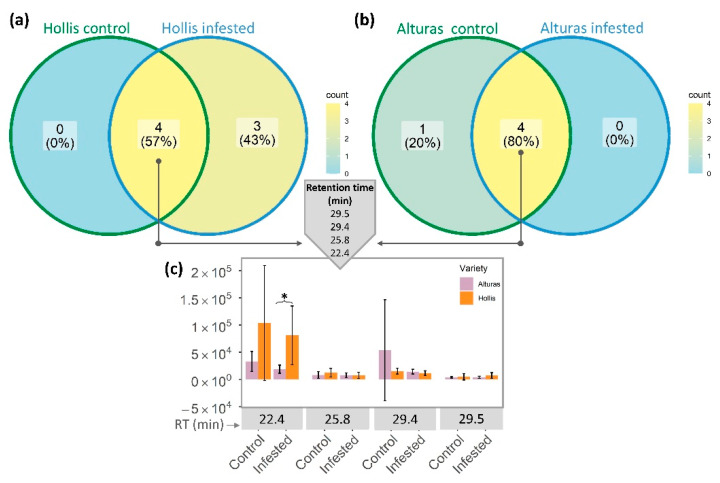
Venn diagram showing common and unique peaks represented by different retention times extracted from GC-FID data of samples from resistant and susceptible wheat infested with Hessian fly. Comparison of (**a**) number of peaks from Hollis infested and control wheat data, (**b**) number of peaks from Alturas infested and control wheat data, and (**c**) peak intensity of four common retention times between both cultivars and treatments. * Significant differences from *t*-test analysis (*p*-value < 0.05).

The number of common and unique VOC peaks from Hessian fly-infested and control plants and resistant (Hollis) and susceptible (Alturas) wheat plants are shown by Venn diagrams (Figure 5). Infested Alturas plants did not show unique peaks, while infested Hollis plants released three unique peaks (Figure 5a), which suggests these three unique RTs can be used as biomarkers to detect resistance to Hessian fly. In our recent study [24], we evaluated VOCs emission profile to assess pea plant responses (resistant and susceptible cultivar) to Aphanomyces root rot. Similar results were found with more peaks found in infected than control samples. In another wheat study [30], a higher concentration of volatiles was observed in wheat cultivar (Lambert) susceptible to the *Barley yellow dwarf luteovirus* in comparison to transgenic-resistant genotype and control plants, which could be associated with plant responses to aphid vector (*Rhopalosiphum padi* L.).

**Figure 6:**
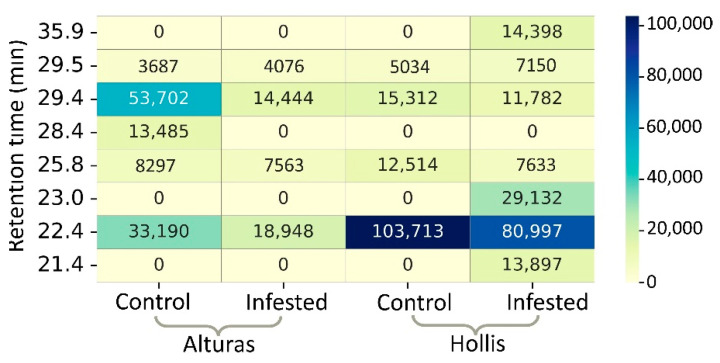
Heat-map showing the peak intensity extracted from GC-FID data of samples from resistant and susceptible wheat infested with Hessian fly.

In this study, interestingly, both plant cultivars exhibited the same four common peaks (Figure 5a,b). The greatest average peak intensity (*p*-value *<* 0.05) was found at 22.4 min RT where Hollis infested samples showed 4.3 times higher average peak intensity than Alturas infested samples (Figure 5c). Since this peak was also found to be higher in Hollis control samples, further investigations may be needed. Heatmap shows that the three induced VOC peaks from Hollis-infested samples were at 21.4, 23.0, and 35.9 RTs (Figure 5a and Figure 6).

## 4. Conclusions

In both potato and wheat studies, interestingly the resistant cultivars released a greater number of VOCs (peak RTs). For example, at 2 DAI, SB22 plants infested with Mc1 showed a higher number of induced VOCs. Additionally, at 25 DAI, constitutive VOCs of SB22 plants inoculated with Mc1 displayed a higher average peak intensity between 1.6–9.5 times than those of the control samples. Similarly, samples from Hollis only showed induced VOCs in infested samples (induced VOCs were absent in Alturas). The reported studies highlight the differences in VOC emission profiles between healthy (control) and infected/infested samples, as well as profile differences between resistant and susceptible cultivars. With this potential, VOC emission profiles can serve as a phenotyping tool to screen the plant materials non-invasively, especially at early time points. Further studies are required to determine changes in VOC emission profiles associated with diverse genes providing resistance to Hessian fly in multiple wheat cultivars.

The major benefit would be the screening of the same plant materials across several time points. To identify the compounds associated with peak RTs, analyses of samples using gas chromatography-mass spectrometer, and comparison with the National Institute of Standards and Technology (NIST) database is needed. Even so, the identification of compounds associated with VOC peak RTs remains challenging (e.g., [24,25]). In this regard, the VOC peaks or VOC emission profiles [31] can be used as biomarkers. Several high-throughput sensing techniques such as e-noses and IMS-based systems can further increase the throughput in sampling, data acquisition, and analysis [31]. Our previous study investigated a high-throughput VOC sensing system, a field asymmetric ion mobility spectrometer, with successful results for post-harvest potato rot detection for applications in storage [32,33]. In our future studies, such high-throughput VOC sensing systems will be explored with an increase in the number of samples, replicates, and cultivars under both treatment conditions.

## Data Availability

The data presented in this study are available on request from the corresponding author.
